# Enhanced yield of oleuropein from olive leaves using ultrasound‐assisted extraction

**DOI:** 10.1002/fsn3.654

**Published:** 2018-04-17

**Authors:** Domenico Cifá, Mihaela Skrt, Paola Pittia, Carla Di Mattia, Nataša Poklar Ulrih

**Affiliations:** ^1^ Faculty of Bioscience and Technology for Food, Agriculture and the Environment University of Teramo Teramo Italy; ^2^ Department of Food Science and Technology Biotechnical Faculty University of Ljubljana Ljubljana Slovenia; ^3^ Centre of Excellence for Integrated Approaches in Chemistry and Biology of Proteins (CipKeBiP) Ljubljana Slovenia

**Keywords:** extraction efficiency, maceration, oleuropein, olive leaves, ultrasound

## Abstract

The aim of this study was to optimize the extraction of oleuropein from olive leaves through a systematic study of the effects of different parameters of ultrasound‐assisted extraction (USAE) on the oleuropein yield, in comparison with conventional maceration extraction. A range of operational parameters were investigated for both conventional maceration extraction and USAE: solvent type, olive leaf mass‐to‐solvent volume ratio, and extraction time and temperature. Oleuropein yield was determined using high‐performance liquid chromatography, with total phenolics content also determined. The optimized conditions (water–ethanol, 30:70 [v/v]; leaf‐to‐solvent ratio, 1:5 [w/v]; 2 hr; 25°C) provided ~30% greater oleuropein extraction yield compared to conventional maceration extraction. The total phenolics content obtained using the optimized USAE conditions was greater than reported in other studies. USAE is shown to be an efficient alternative to conventional maceration extraction techniques, as not only can it offer increased oleuropein extraction yield, but it also shows a number of particular advantages, such as the possibility of lower volumes of solvent and lower extraction times, with the extraction carried out at lower temperatures.

## 1 INTRODUCTION

In recent years, the agronomic, cosmetic, and pharmaceutical industries have increased their interest in natural extracts from plants and by‐products that are rich in bioactive compounds. This is due to the need to meet the growing demand for natural preservatives and to produce novel functional foods with significant health benefits (Joana Gil‐Chávez et al., 2013; Ribeiro, Estanqueiro, Oliveira, & Sousa Lobo, 2015; Soler‐Rivas, Espín, & Wichers, 2000). In this respect, olive leaves are a promising vegetable matrix, from which natural extracts can be obtained that are rich in a large variety of bioactive compounds, as they are rich in phenolic compounds. The main constituents of olive leaves are secoiridoids (e.g., oleuropein, ligstroside, dimethyloleuropein) and flavonoids (e.g., apigenin, kaempferol, luteolin), along with other phenolic compounds (e.g., hydroxytyrosol, tyrosol, caffeic acid).

Oleuropein is the most representative polyphenolic constituent of olive leaves, as the compound responsible for the bitterness of both table olives and extra‐virgin olive oil. Several studies have shown a wide variety of *in vitro* and *in vivo* properties for oleuropein, including antioxidant, antiviral, antibacterial, and anti‐inflammatory activities (Alzweiri & Al‐Hiari, 2013; Hayes, Allen, Brunton, O'grady, & Kerry, 2011; Peralbo‐Molina & de Castro, 2013). In recent years, attention has been focused also on the technological aspects of oleuropein, especially its use in emulsified food matrices. According to its amphiphilic structure, oleuropein has been shown to have surface activity and influences the emulsification process in model systems (Di Mattia, Sacchetti, & Pittia, 2011; Di Mattia et al., 2014; Souilem, Kobayashi, Neves, Jlaiel, et al., 2014; Souilem, Kobayashi, Neves, Sayadi, et al., 2014) and in complex formulations (Di Mattia et al., 2015; Giacintucci, Di Mattia, Sacchetti, Neri, & Pittia, 2016). Oleuropein has also been shown to inhibit oxidative phenomena in heterophasic systems (Paradiso et al., 2016).

Studies over the last few years have focused on extraction techniques and processing methods, to determine those that are most suited to obtain natural extracts for commercial applications. The extraction of bioactive compounds from plants is usually carried out using conventional techniques, such as maceration. This, however, can result in the loss of some compounds, low extraction yields, long extraction times, and high consumption of energy.

A recent review highlighted the importance of olive leaves in this context, defining their composition and preparation methods, and the application of emerging technologies for recovery of bioactive compounds from this matrix (Souilem et al., 2017). The recent literature indicates an increasing focus on research on olive leaves, which has confirmed their great potential as a valuable material in various fields. However, more studies are needed to optimize their extraction conditions to increase the yield of bioactive compounds, by decreasing the extraction costs and preserving their functional activities. In this sense, ultrasound‐assisted extraction (USAE) is considered a novel technique that can be used to intensify slow processes, such as the leaching of polyphenols from vegetable matrices. USAE thus has the potential to reduce extraction times and extraction solvent volumes, and to increase recoveries of active compounds.

The application of ultrasound waves produces intense pressure and temperature gradients within the material, which can induce physical structural disruption due to cavitation. This then enhances mass transfer and release of intracellular substances into the extraction medium. USAE with olive leaves has been reported previously in studies of the effects of different process parameters on the extraction kinetics and the composition of extracts, such as the electric amplitude, the emitter surface, and temperature (Ahmad‐Qasem et al., 2013; Chemat, Tomao, & Virto, 2008; Esclápez, García‐Pérez, Mulet, & Cárcel, 2011; Luque‐Garcıa & De Castro, 2003).

The aim of this study was to maximize the extraction yield of oleuropein from olive leaves and to decrease operating costs with the possibility of lower volumes of solvent and lower extraction times and temperatures. In that respect, we investigate the application of USAE and the different extraction parameters such as type of solvent, ratio of olive leaf mass to solvent volume, and extraction time and temperature. For comparison, parallel extractions were carried out using maceration.

## 2 MATERIALS AND METHODS

### 2.1 Chemicals

Oleuropein (purity by HPLC, ≥80%) was from Sigma‐Aldrich Fine Chemicals (St. Louis, MO, USA). All of the other chemicals used were of analytical grade. Acetonitrile (Chromasolv gradient grade for HPLC), acetic acid, ethanol, and hydrochloric acid were from Sigma‐Aldrich. Sodium hydroxide was from Merck Chemicals. All solutions were prepared with ultrapure or reverse osmosis water from a Millipore RIOS 5 purification system (Bedford, MA, USA).

### 2.2 Plant materials

Leaves of the olive (*Olea europaea* L.) variety “*Istrska belica”* were collected from Orchard Školarice (Istra, Slovenia). The olive leaves were air‐dried at 25°C for 7 days and then ground using a two‐step process. First, they were manually ground using a mortar and pestle, to obtain a coarse powder. This was then processed using a ball mill (MM400; Retsch GmbH, Haan, Germany) to obtain a fine powder. Aliquots of 6 g of the coarse powder samples were processed in the grinding jar with four grinding balls (diameter, 15 mm) and milled at 30.0 Hz for 60 s. The powder was stored until analysis in the dark at room temperature and under low relative moisture. The same sample batch was used throughout the experimental plan.

### 2.3 Extraction procedures

The conventional maceration extraction was carried out in thermostated water bath with rotational agitation (WB‐30 STE; Kambič, Slovenia) at 160 rpm. The USAE was carried out in an ultrasonic bath (frequency, 35 kHz; power, 60/120 W; Sonorex TK 52; Bandelin electronic, Berlin, Germany). The following extraction parameters were examined: solvent composition (100% milli‐Q water, 30%, 50%, 70% [v/v] aqueous ethanol), olive leaf mass‐to‐solvent volume ratio (g/ml; 1:3 to 1:10), and extraction time (maceration, 60–1440 min; USAE, 5–360 min) and temperature (maceration, 25–60°C; USAE, 10–70°C). During the extraction, the water bath temperature was continuously monitored and adjusted, with the extractions carried out in the dark. The olive leaf dispersions were included in plastic conical centrifuge tubes that were fully immersed in the water bath during the extractions. The samples were then centrifuged (Centric 322A; Domel, Železniki, Slovenia) at 2540 × *g* for 10 min. After centrifugation, the supernatants were filtered using 0.45‐μm syringe filters (Minisart RC 15; Sartorius, Germany) and immediately analyzed by HPLC.

### 2.4 Extraction yields

The extraction yields for oleuropein are expressed as mg oleuropein per g initial dry olive leaf and calculated according to Equation (1):

(1) Extraction yield=(oleuropein)/(leaves used in extraction)

### 2.5 Determination of oleuropein in olive leaf extracts

#### 2.5.1 Chromatographic conditions

All solutions of oleuropein (i.e., standards, extracts) were analyzed using an HPLC system (Agilent 1260; Agilent Technologies, Inc., Wilmington, Germany) with a binary pump (G1312B, Infinity), an autosampler (G1367E), and a diode array detector (G4212B). Data signals were acquired and processed on a PC running the dedicated analysis system (Agilent HPLC 2D ChemStation SW; Agilent Technologies, Inc.). The HPLC analysis was carried out using a C18 column (Zorbax Eclipse Plus; 4.6 × 150 mm; 3.5‐μm particle size; Agilent Technologies, Inc.) and a C18 analytical guard column (Agilent Eclipse XDB‐C18; 4.6 × 12.5 mm; 5‐μm particle size).

The separation conditions for oleuropein were as follows: column temperature, 25°C; injection volume, 10 μl; flow rate, 0.3 ml/min. The separation was carried out in gradient mode with a discontinuous gradient of mobile phases A (1% CH_3_COOH) and B (100% acetonitrile): 0–16 min, 5%–15% B; 16–37 min, 15%–30% B; 37–50 min, 30%–40% B; 50–58 min, 40%–50% B; 58–60 min, 50%–100% B; 60–61 min, 100% B; 61–62 min, 100%–5% B; re‐equilibration, 62–70 min, 5% B. The chromatograms were recorded in the range of 200 nm to 600 nm, with the signals for oleuropein seen at 280 nm. The data were processed using the ChemStation Agilent Technologies software. The oleuropein in the olive leaf extracts was identified according to its retention time and peak UV spectra in the extract chromatogram in comparison with the peak of the oleuropein standard. Quantitation was carried out using calibration curves (see below). The data are expressed as means of the mg oleuropein per g dry olive leaf.

#### 2.5.2 Calibration curve

To determine the concentrations of oleuropein in the olive leaf extracts, calibration curves were prepared from stock solutions of oleuropein (800 μg/ml). The standard solutions of 8, 24, 40, 64, and 80 μg/ml oleuropein were prepared by diluting the stock standard with HPLC mobile phase (5% B). Each point of the calibration curve was repeated three times. The *R* ^2^ correlation coefficient was 0.999. The limit of quantification and the limit of detection were calculated on the basis of the standard deviations of the responses and the slopes obtained from the linearity plots of the oleuropein standard solutions. The limits of quantification and detection were calculated as 3.3α/S and 10α/S, respectively, where α is the standard deviation of the y‐intercept and S is the slope of the regression line. The limits of quantification and detection were 5.03 μg/ml and 1.66 μg/ml, respectively. The oleuropein yields were computed, with the mean value of 99.9% determined.

### 2.6 Oleuropein stability

The stability of pure oleuropein and the oleuropein in the olive leaf extracts at different pH values (2,3, 4, 5, 8, 10) and in 70% ethanol was followed over time using HPLC. All of the samples were diluted with HPLC mobile phase (5% B) prior to the HPLC analysis, which was run as described above.

### 2.7 Determination of total phenolics content

Analyses were carried out on a limited number of extract samples to determine the overall content of the phenolic compounds in the olive leaf extracts, to complement the HPLC analysis and the data on the bioactive compound of interest (i.e., oleuropein). There, total phenolic contents (TPCs) of the olive leaf extracts were estimated as gallic acid equivalents (GAE), expressed as mg gallic acid per g dry matter (dm), according to the Folin–Ciocalteu method, following the procedure of Gutfinger (1981). The TPCs of the extracts were measured using a UV‐visible spectrophotometer (Hewlett Packard 8453; Agilent Technologies, Mulgrave, Vic., Australia), with the absorbance measured at 765 nm.

### 2.8 Statistical analysis

All of the experiments were performed in triplicate, and the data are given as means ±standard deviation (*SD*). The statistical significances of the process parameters were evaluated using analysis of variance (ANOVA), and Tukey's tests were performed using the XLSTAT software, to establish the significances of the differences among the means at the 95% of confidence level, where *p *<* *.05 is regarded as significant.

## 3 RESULTS AND DISCUSSION

The operational parameters of solvent system, ratio of olive leaf mass to solvent volume, and extraction time and temperature were studied in distinct experiments, with a view to improve the oleuropein yield from olive leaves using USAE, and in comparison with the conventional maceration method.

### 3.1 Maceration extraction factors

#### 3.1.1 Solvent composition

**Figure 1:**
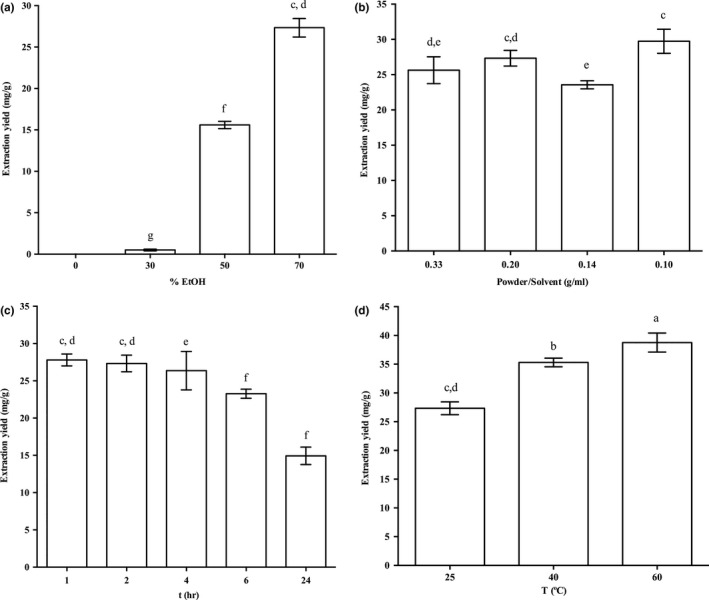
Effects of different parameters on the oleuropein extraction yields from dry olive leaves using the conventional maceration extraction. (a) Influence of increasing ethanol in the solvent on the oleuropein extraction yield, with a 1:5 (w/v) ratio of fresh leaf mass to solvent volume, extracted for 120 min at 25°C. (b) Influence of the ratio of dry leaf mass to solvent volume on the oleuropein extraction yield, with 70% aqueous ethanol, extracted for 120 min at 25°C. (c) Influence of extraction time on the oleuropein extraction yield, with 70% aqueous ethanol, a 1:5 (w/v) ratio of dry leaf mass to solvent volume, extracted at 25°C. (d) Influence of extraction temperature on the oleuropein extraction yield, with 70% aqueous ethanol, a 1:5 (w/v) ratio of dry leaf mass to solvent volume, extracted for 120 min

The polarity of the solvent has an important role in the selective extraction of phenolic compounds, with ethanol, methanol, acetone, and aqueous ethanol solutions as the most commonly used solvents for their extraction (Altıok, Bayçın, Bayraktar, & Ülkü, 2008; Naczk & Shahidi, 2006). At a constant solid‐to‐liquid ratio of olive leaves to solvent volume of 1:5 (w/v), and maceration time (120 min) and temperature (25°C), the oleuropein yield increased according to the increasing ethanol content in the extraction solvent (Figure 1a). Thus, the greatest oleuropein yield was obtained here using 70% aqueous ethanol (27.3 ± 1.1 mg/g). The use of water alone did not result in any detectable signal across these conditions for oleuropein yield quantification. The pH of the water was also varied (2.0, 3.0, 4.0, 5.7) by keeping constant the solid‐to‐liquid ratio (1:5) and the extraction time (2 hr) and temperature (25°C), to evaluate any effects of acidification of this aqueous extraction. However, no significant differences were observed with respect to the reference pH (5.7). These data thus confirmed that water is not a good solvent to extract oleuropein from olive leaves.

This is in agreement with other literature data that have shown that mixtures of organic solvents can lead to higher oleuropein recoveries compared to pure solvents (Yateem, Afaneh, & Al‐Rimawi, 2014), especially for the use of 70% aqueous ethanol (Stamatopoulos, Chatzilazarou, & Katsoyannos, 2013). Some studies have also reported that along with other flavonoids, oleuropein is a main component of ethanolic extracts of olive leaves, while the levels decrease in water extracts, where a prevalence of hydroxytyrosol glucoside and phenolic acids has been reported (Herrero et al., 2011; Lee et al., 2009; Quirantes‐Piné et al., 2013). The highest extraction yield obtained using 70% aqueous ethanol is due to its chemical properties. The ethanol molecule contains a hydrophilic hydroxyl group that is available in hydrogen bond to water molecules, and an alkyl chain that confers a degree of hydrophobicity to the system in which it is present. The properties, effects, and mode of action of ethanol are affected by the ratio of water to ethanol, which in turn affects the solution properties of the mixture (Parke & Birch, 1999), and thus its solubilization of amphiphilic compounds, like oleuropein in this study. Combined with its molecular affinity for phenolic compounds, these properties make 70% aqueous ethanol the best extraction mixture for increased oleuropein yield from the olive leaves. This solvent of 70% aqueous ethanol was thus chosen and used throughout the subsequent experiments.

#### 3.1.2 Dry olive leaf mass‐to‐solvent ratio

The impact of the solid‐to‐liquid ratio (i.e., ratio of dry olive leaf mass to solvent volume) on the extraction of oleuropein from these olive leaves was also tested. The ratios used were 1:3, 1:5, 1:7, and 1:10 (w/v; 0.33, 0.2, 0.14, 0.1 mg/ml, respectively) with the extraction time of 2 hr and temperature of 25°C (Figure 1b). Here, the 1:10 ratio provided the greatest oleuropein extraction yield (29.7 ± 1.7 mg/g), while no significant differences were seen between the other ratios tested. This increase in the oleuropein yield obtained through modulation of the olive leaf mass‐to‐solvent ratio is in agreement with mass transfer principles, as a lower solid‐to‐liquid ratio will provide a higher driving force for the extraction, in agreement with İlbay, Şahin, and Büyükkabasakal (2014) for the same matrix.

Moreover, and as expected, if the data are expressed as the concentration of oleuropein obtained in the extract (g oleuropein/ml), a decrease in the oleuropein concentration with the increase in the solid‐to‐liquid ratio was noted as a consequence of a dilution effect (Stamatopoulos et al., 2013).

#### 3.1.3 Extraction time

The optimal extraction time was investigated for the extraction of the olive leaf at constant solvent composition (70% aqueous ethanol), ratio of olive leaf mass to volume (1:5; w/v) and temperature (25°C), with extraction times up to 24 hr. These data showed that the extraction yield does not change significantly over the first 4 hr of extraction, with oleuropein content obtained within 1 h being the highest numerically (27.8 ± 0.8 mg/g; Figure 1c). Moreover, after 4 hr, the extraction yield decreased significantly with increased extraction time, which was probably due to oxidative decomposition of oleuropein, as previously reported in other studies (Ansari, Kazemipour, & Fathi, 2011; Malik & Bradford, 2008).

**Figure 2:**
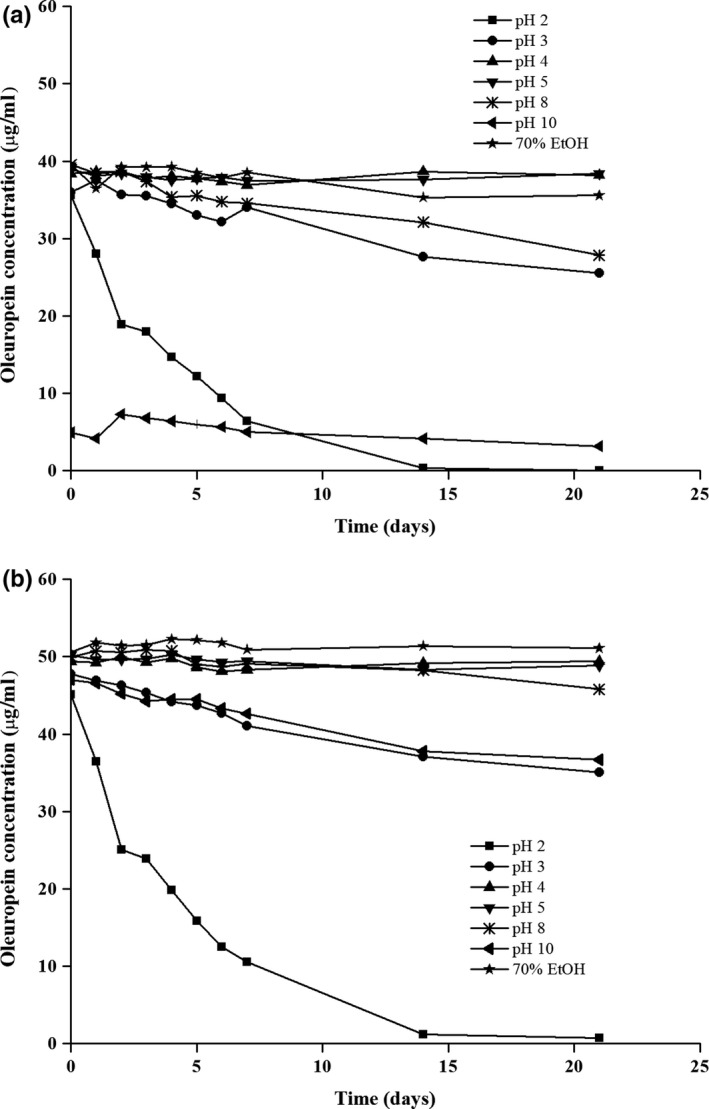
Stability of pure oleuropein (a) and oleuropein in the olive leaf extract (b) in aqueous systems at different pH values and in 70% aqueous ethanol

In addition, to support this hypothesis further, the changes with time in the concentrations of both pure oleuropein (Figure 2a) and the oleuropein in the olive leaf extract (Figure 2b) were tested in aqueous systems at different pH values (2, 3, 4, 5, 8, 10) and in 70% ethanol under constant temperature (25°C). Here, at pH 4 and 5, no significant changes in the oleuropein concentrations were seen over 21 days, thus with little or no degradation detected. In contrast, there were similar decreases in the oleuropein concentrations at the more extreme pH for both the pure form (Figure 2a) and the olive leaf extract (Figure 2b). In the systems at pH 2, 3, and 8, hydroxytyrosol was detected, with oleuropein as the main compound, while in the 70% aqueous ethanol another peak in the HPLC chromatogram was detected, which was attributed to oleuropein aglycon (data not shown). These data lead to the conclusion that oleuropein degradation under these chosen experimental conditions is mostly due to its hydrolysis.

#### 3.1.4 Extraction temperature

The temperature influence on the oleuropein extraction yield was studied at constant solvent composition (70% aqueous ethanol), solid‐to‐liquid ratio (1:5), and time (120 min). Increased oleuropein yield was seen with increasing temperature, which would appear to be related to increased solubility and diffusion coefficients (Figure 1d). However, while from 25°C to 40°C this increase in oleuropein yield was significant, a further increase in the temperature from 40°C to 60°C provided only a limited (and nonsignificant) improvement in oleuropein yield. This might be attributed to the simultaneous occurrence of events that can counteract the higher oleuropein mass transfer from the olive leaves to the extraction medium, including its thermal degradation. Indeed, some studies have highlighted relatively low thermal stability of oleuropein (Ahmad‐Qasem et al., 2013; Cacace & Mazza, 2003; Dent et al., 2013; Jokić et al., 2010; Stamatopoulos et al., 2013; Yateem et al., 2014). However, 60°C was found optimal temperature as extraction condition in some papers that studied the effect of cultivar and season on olive leaves phenolic compounds and oleuropein (Blasi, Urbani, Simonetti, Chiesi, & Cossignani, 2016).

### 3.2 Ultrasound‐assisted extraction optimization

Ultrasound‐assisted extraction has been applied to improve yields of thermodegradable and chemically labile compounds, such as tocopherols and vitamin E isomers from *Amaranthus caudatus* seeds (Bruni, Guerrini, Scalia, Romagnoli, & Sacchetti, 2002; Bruni et al., 2001), vitamin C from fruit juices (Akkermans, Wu, & Compton, 1998), and phenolic compounds from pistachio hulls, coconut shells, and strawberries (Goli, Barzegar, & Sahari, 2005; Herrera & De Castro, 2004, 2005; Rodrigues & Pinto, 2007).

To optimize the oleuropein extraction yields with the addition of USAE here, several operational parameters were again studied, including solvent composition, olive leaf‐to‐solvent volume ratio, and extraction time and temperature. Table 1 shows the operational conditions applied during this oleuropein extraction from olive leaves by USAE, along with the corresponding yields. It can be seen that under these conditions, the solvent composition was the sole parameter that significantly affected the oleuropein extraction. Moreover, as already observed without USAE, the oleuropein yields from the olive leaves increased with increasing ethanol concentration in the solvent. Indeed, again the highest oleuropein yield was obtained with 70% aqueous ethanol (38.1 ± 1.8 mg/g), with these data in agreement with other studies (Hemwimol, Pavasant, & Shotipruk, 2006).

**Table 1:** Ultrasound‐assisted extraction experimental parameters and corresponding oleuropein yields

Solvent conditions		Incubation conditions		Oleuropein
Solvent (% ethanol)	Solid:liquid^a^ (g/ml)	Time (min)	Temperature (°C)	Yield (mg/g leaves)^b^
0	0.2	120	25	n.a.
30	0.2	120	25	3.1 ± 0.1^d^
50	0.2	120	25	10.8 ± 0.6^c^
70	0.2	120	25	38.1 ± 1.8^a^
70	0.2	5	25	37.6 ± 0.6^a^
70	0.2	15	25	36.2 ± 0.4^a^
70	0.2	30	25	36.1 ± 1.0^a^
70	0.2	60	25	35.6 ± 1.1^a,b^
70	0.2	120	25	38.1 ± 1.8^a^
70	0.2	240	25	37.5 ± 1.8^a^
70	0.2	360	25	36.7 ± 1.6^a^
70	0.33	120	25	36.8 ± 2.1^a^
70	0.2	120	25	38.1 ± 1.8^a^
70	0.14	120	25	38.8 ± 1.5^a^
70	0.1	120	25	31.6 ± 0.6^b^
70	0.2	120	10	38.3 ± 0.8^a^
70	0.2	120	25	38.1 ± 1.8^a^
70	0.2	120	70	39.2 ± 0.9^a^

n.a., not applicable.

Different superscript letters in the same column indicate that difference is statistically significant (Tukey's tests; *p* < .0001).

a Ratio of dry leaf mass to solvent volume.

b Leaf mass expressed as dry matter.

When 70% aqueous ethanol was used as the solvent here, none of the other operational extraction parameters (i.e., solid‐to‐liquid ratio, extraction time and temperature) influenced the oleuropein yield. The oleuropein yield including USAE ranged from 35.6 ± 1.1 mg/g to 39.2 ± 0.9 mg/g. The only exception here was for the 1:10 leaves‐to‐solvent ratio (Table 1), with a lower extraction yield (reduced by ~32%), although it remained slightly higher than that obtained by conventional maceration. Overall, these data confirm the higher efficiency of oleuropein extraction under USAE, as related to the mechanisms of action on the bulk extraction system.

**Figure 3:**
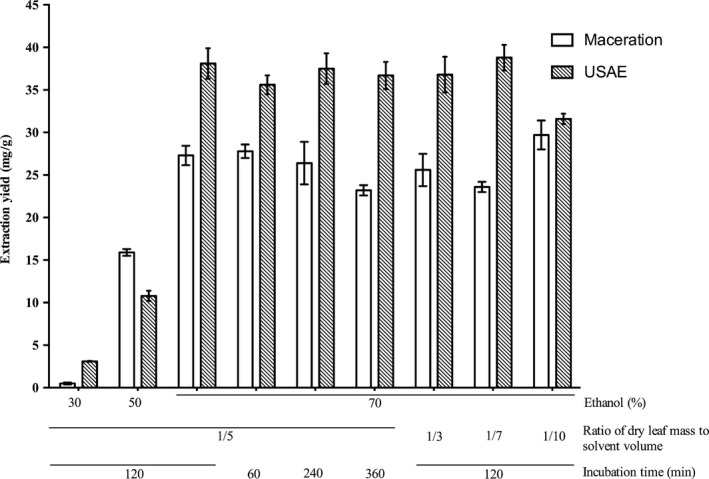
Comparison of the conventional maceration extraction and USAE under the same operational parameters, with all carried out at 25°C. (a) 30% aqueous ethanol (solvent), 1/5 (w/v; ratio of dry leaf mass to solvent volume), 120 min (extraction time). (b) 50% ethanol, 1/5 (w/v), 120 min. (c) 70% ethanol, 1/5 (w/v), 120 min. (d) 70% ethanol, 1/5 (w/v), 60 min. (e) 70% ethanol, 1/5 (w/v), 240 min. (f) 70% ethanol, 1/5 (w/v), 360 min. (g) 70% ethanol, 1/3 (w/v), 120 min. (h) 70% ethanol, 1/7 (w/v), 120 min. (i) 70% ethanol, 1/10 (w/v), 120 min

Figure 3 shows the extraction yields obtained by conventional maceration and USAE under the same operational parameters. These data demonstrate that USAE provides significantly higher yields of oleuropein from milled olive leaves compared to maceration (from 6% to 83.9% increases), except for run *b* (Figure 3; 50% aqueous ethanol, 1/5 [w/v], 120 min, 25°C). These data are also confirmed by the TPCs obtained from the Folin–Ciocalteu assay carried out on the same samples. For run c (Figure 3), for instance, the TPCs of the maceration extracts and USAE were 32.7 mg GAE/g dm and 138.4 mg GAE/g dm, respectively. Based on the substantial differences observed between USAE and maceration extracts in TPC content, it is reasonable to assume that USAE extract contains many other phenolics in higher concentrations besides the oleuropein.

These data highlight that when using USAE and an aqueous solvent of 70% ethanol, the extraction time can be reduced to 5 min (Table 1), which produced a yield of 37.6 ± 0.6 mg oleuropein/g olive leaf. Instead, with maceration, the highest yield that was obtained here with a 1‐hr extraction time (27.8 ± 0.8 mg/g) was still lower than that obtained by USAE with the much shorter extraction (Figure 1c).

The solid‐to‐liquid ratio results indicate that by using USAE, the ratio 1:3 (w/v) was sufficient to extract high quantities of oleuropein (Table 1); on the contrary, the maximum quantity of oleuropein that was extracted by maceration was using the 1:10 ratio (Figure 1b). Finally, the oleuropein extraction efficiency of USAE was not significantly influenced by temperature, allowing the extraction to be performed at room temperature, thereby avoiding degradation of the bioactive compounds during the extraction.

To determine the overall value of this optimized USAE extraction method, these data for oleuropein yield expressed in terms of TPCs of extracts were compared with TPC data from other studies in the literature (Table 2). Here, these TPC data obtained in this study using USAE are among the highest yields of extractions, at 138.4 mg GAE/g dm. These comparisons included other extraction techniques like microwave‐assisted extraction (Şahin et al., 2017), steam explosion (Romero‐García et al., 2016), and pressurized liquid extraction (Herrero et al., 2011). To the best of our knowledge, similar yields were obtained by Difonzo et al. (2017), for both water and aqueous ethanol extractions, although only when hot‐dried olive leaves were used as the starting materials. On the contrary, the same authors reported lower oleuropein content for the same extract than that determined in this study (21.31 ± 0.53 mg/g) which again supports the use of USAE for the extraction of this bioactive compound, oleuropein.

**Table 2:** Review of total phenolic contents from olive leaves reported according to different extraction methods

Extraction technique	Extraction solvent	Total phenolic content (mg GAE/g dm)	References
Ultrasound‐assisted extraction^a^	70% ethanol	138.4	Present study
Maceration^b^	70% ethanol	32.7	Present study
Maceration (hot air‐dried leaves)	70% ethanol	138.0	Difonzo et al. (2017)
Eco‐friendly deep eutectic solvent	Glycerol:glycine:water (7:1:3)	106.3	Athanasiadis, Grigorakis, Lalas, and Makris (2017)
Low‐transition temperature mixture	Glycerol, sodium acetate	53.8	Karageorgou, Grigorakis, Lalas, and Makris (2017)
Solvent‐free microwave‐assisted extraction	Water as pretreatment	0.0025	Şahin et al. (2017)
Maceration (freeze‐dried leaves)	70% ethanol	108.0	Difonzo et al. (2017)
Maceration	60% ethanol	66.6	Blasi et al. (2016)
Instant controlled pressure drop	95% ethanol	67.8	Mkaouar, Gelicus, Bahloul, Allaf, and Kechaou (2016)
Steam explosion	Saturated steam	20.9	Romero‐García et al. (2016)
Maceration	80% ethanol	66.0	Ahmad‐Qasem et al. (2014)
Ultrasound‐assisted extraction	80% ethanol	66.0	Ahmad‐Qasem et al. (2014)
Heated water/ glycerol mixtures	9.3% Glycerol	51.9	Apostolakis, Grigorakis, and Makris (2014)
Maceration	Methanol	40.0	Alzweiri and Al‐Hiari (2013)
Maceration	70% ethanol	144.2	Salah, Abdelmelek, and Abderraba (2012)
Maceration	80% Acetone	24.9	Abaza et al. (2011)
Pressurized liquid extraction	Water	58.7	Herrero et al. (2011)

GAE, gallic acid equivalents; dm, dry matter.

a Extraction parameters: 70% ethanol, 25°C, 1:5 (w/v), 10 min.

b Extraction parameters: 70% ethanol, 25°C, 1:5 (w/v), 120 min.

However, it is also evident that this comparison needs to consider the influence of other factors that might affect the oleuropein and phenolic compounds in olive leaf extracts, including the olive cultivar, leaf color/ age, time (season) of collection, and the drying and storage conditions (Afaneh, Yateem, & Al‐Rimawi, 2015; Blasi et al., 2016; Ranalli et al., 2006; Wang, Gao, Ye, Chen, & Jiang, 2008).

## 4 CONCLUSIONS

The present data show that the oleuropein extraction yield from olive leaves by the conventional maceration method is significantly affected by the operational parameters of the solvent, the solid‐to‐liquid ratio, and the time and temperature of the extraction. Therefore, to obtain relatively high amounts of this bioactive compound, there is the need for optimization of the extraction method. On the contrary, USAE is shown to be an efficient alternative to conventional extraction techniques, as not only can it offer increased oleuropein extraction yield, but it also shows a number of particular advantages, such as the possibility of lower volumes of solvent and lower extraction times, with the extraction carried out at lower temperatures. All of these advantages will contribute to decreased operating costs and environmental issues. However, to understand the action of US, the ultrasonic energy introduced in their system should be determined as well as the effect of US power on the extraction efficiency.

## CONFLICT OF INTEREST

None declared.

## AUTHOR CONTRIBUTIONS

The authors declare no conflict of interests. N.P.U, P.P., C.DM., and M.S. involved in conception or design of the work; D.C. and M.S. involved in data collection; D.C., M.S., and C.DM. contributed to data analysis and interpretation; D.C. drafted the article; P.P., C.DM., M.S., and N.P.U. involved in critical revision of the article; N.P.U. contributed to the final approval of the version to be published.
